# Supplementary material for: Chromothripsis during telomere crisis is independent of NHEJ, and consistent with a replicative origin
Source: Genome Res. 2019 May;29(5):737–49. doi: 10.1101/gr.240705.118 (PMC6499312; doi:10.1101/gr.240705.118)
Supplement: Supplemental Material [file supp_gr.240705.118_Supplemental_file_1.zip › contigs/annotated_contigs/DB53/contig.2.DB53_length_891_mean_cov_7.90684624018.docx]

**DB53_length_891_mean_cov_7.90684624018**

```
GCGGCCGCCCGGACATCAGTGAGCGGCTCATCAGCGCCTCCCTCTTCCTGCGCTTCCTCTGCCCAGCCATCATGTCGCCCTCACTCTTC
 >chr9:124528831-124529292 + E=2e-262 p=0e+00
AACCTGCTGCAGGAGTACCCTGATGACCGCACTGCCCGCACCCTCACCCTCATCGCCAAGGTCACCCAGAACCTGGCCAACTTTGCCAA

GTGAGTGCCTCCTCCCTCACCAGGCAGAGTTGGGCAGGGCTGGTGTCCACAGGGCAGGCCCTGGGGGTGGTTCTGCCCCCAAGCTGAGC

AGAGACCATAAACAGGCCCTGGTTTTGTCCCTGTCATCCTCAGTCCTTCTCCTTCCCGCTTTTTAAAAAATAAAATCTCCACACCCTGT

CCTTCTACTCAAGGTATATGTCATTTTGGTATGTTTCTTAATTTTTAAAGGTGACGACATGAGACATTTTTTTCCTTCTGCTTTTTTGA

ATTAATGTATTATTAT|TGAATTAATGTATTATTATTGAC|CCAAGAAGTCCTGGGTCAATTACTGATGTCTTAAGACTCGCGTACCTC
                                        >chr9:123199513-123199920 + E=7e-233
ACCCTACCTACCGTTGAGAAATAGCTTTTCCAATTTCTCCAGCAGCTCAGCACATTGATTCAGCTGTTCCTGGAAACCCTCGGCCACAC

AGTAATCCACATCACTGGCCTGCAGCAACTCCTCAAATGCCTTAATCATGGTGTTCATGTGTTGACGGGAGATGACACAGATGCCATGG

CCATCCTTAATCTGCTGCCGTTGAAGGGAGAGCTCCCTGGCTTGGGACTGAACTAATGAATCGTATCTGATAAAAGAGGGGAAAGAAAA

TACTTGGACCATTTTAGAATTGAATTTTTGTGACCTTAACACTAATTGGAAAAATGACAGAGTACAATTTTTAAAAGCCTGCAGCTGTA

CAA
```
